# The Vogt Collection: reactivating a treasure facilitating brain research, neurology and psychiatry

**DOI:** 10.1093/brain/awaf365

**Published:** 2025-10-01

**Authors:** Katrin Amunts

**Affiliations:** Institute of Neuroscience and Medicine, INM-1, Research Centre Jülich, Jülich 52428, Germany; Cécile and Oskar Vogt Institute for Brain Research, Medical Faculty & University Hospital Düsseldorf, Düsseldorf 40225, Germany

## Abstract

Over 125 years ago, Cécile and Oskar Vogt began assembling an extensive collection of brain histological sections and related documents. Katrin Amunts explains how digitizing these materials will connect them with modern neuroscience, creating resources for research spanning basic science, medicine, history and ethics.

***This year marks the 150th anniversary of Cécile Vogt's and the 155th anniversary of Oskar Vogt's births. These two remarkable researchers created a most comprehensive and unique collection of histological sections and other objects—what is now called ‘the Vogt Collection’*.**

To realize their ambitious research goals and to better understand the background against which neurological and psychiatric conditions emerge, the Vogts collected brains and studied them. During their professional lives, which spanned over 60 years, they processed large series of histological sections—altogether about 850 000 sections from different species (Fig. 1).

**Figure 1:**
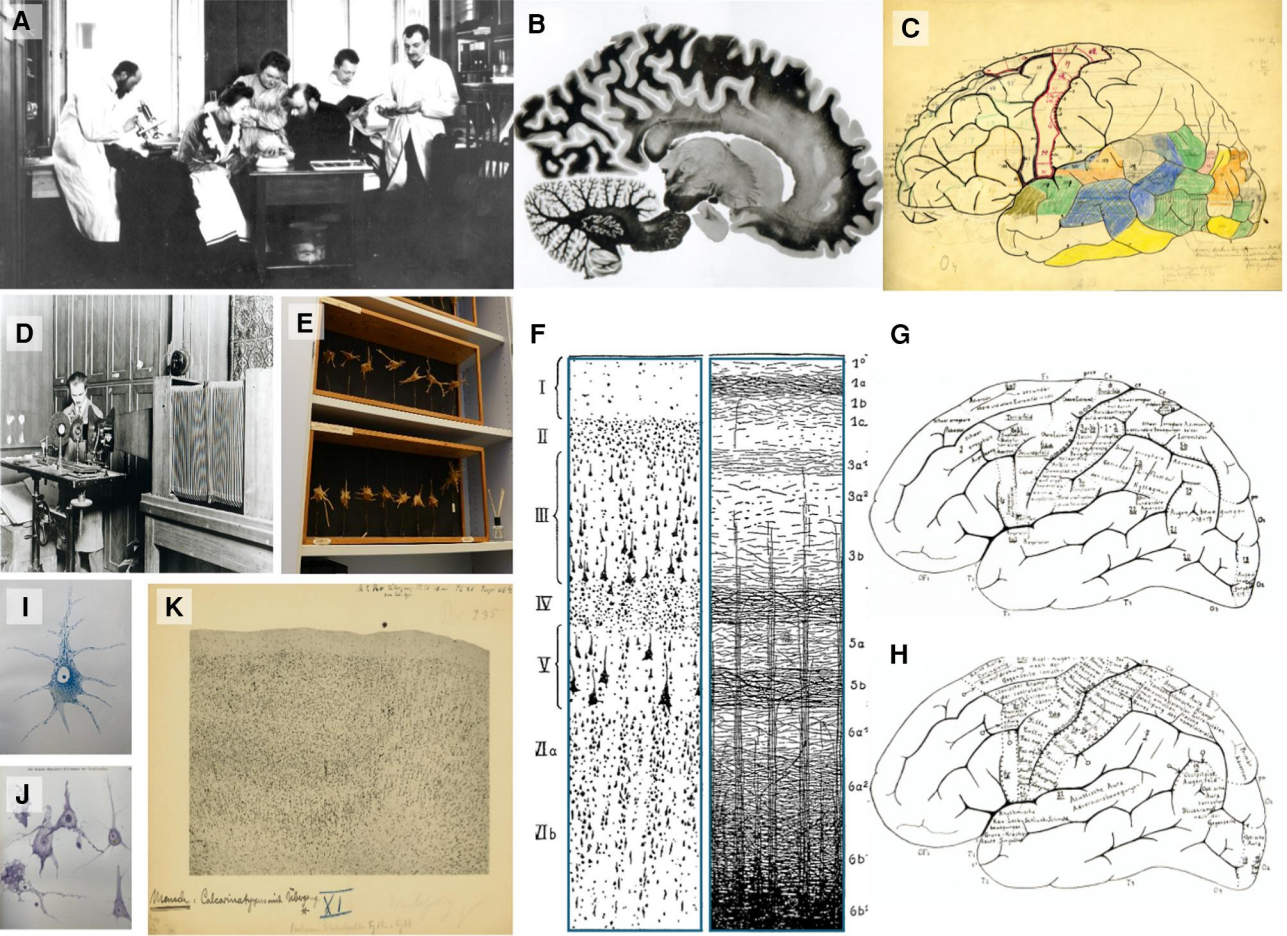
**The Vogts, part of their team and research concept**. (**A**) Cécile and Oskar Vogt (in the *middle*, sitting) together with Korbinian Brodmann (at the window), the technician Louise Bosse, and the scientific collaborators Max Lewandowski and Max Borchert in the ‘Neurobiological Laboratory’ of the University of Berlin (from *left* to *right*) around 1904/05. (**B**) Sagittal, myeloarchitectonic section which formed the basis to develop a myeloarchitectonic map. (**C**) Hand drawn draft of the brain map of the Vogts, with areas labelled and coloured. (**D**) For documentation, the researchers used an optical bank, which allowed high-quality photographs of even single cells and axons. (**E**) Three-dimensional models of neurons that were done by drawing contours of cells at high resolution at different focusing planes and reconstructing them with papier mâché. (**F**) Scheme illustrating the correspondence between cyto- and myeloachitecture.^1^ (**G**) Proposed human homologues of stimulation experiments in monkeys; figure 3 in Vogt and Vogt^2^; and (**H**) stimulation experiments of Otfried Foerster, figure 4 of Vogt and Vogt^2^ projected to the schematic map of figure 3. (**I** and **J**) Progress in staining facilitated cyto- and myeloarchitectonic mapping in the Vogt laboratory: (**I**) the example shows a Nissl-stained pyramidal cell; and (**J**) pathologically altered neurons, Nissl-stained (taken from Spielmeyer, *Histopathologie des Nervensystems. Erster Band: Allgemeiner Teil.* Berlin, Springer, 1922). (**K**) Micrograph of the cytoarchitectonic border between areas 17 (to the *left* of the asterisk-labelled border) and 18 (to the *right* of the asterisk-labelled border) in the human brain annotated by Brodmann. Note the change in the architecture of cells at the border between the two areas: for example, area 17 has neurons with a smaller size, and a more pronounced laminar pattern than area 18. Images from the Archive of the C. & O. Vogt-Institute of Brain Research, University Düsseldorf, Germany.

Cécile Vogt studied medicine in Paris and became internationally renowned for her research into the extrapyramidal system of the brain, neurostimulation and myelinization, among other things. Oskar Vogt's work laid the foundations for an interdisciplinary approach to brain research and the mapping of the brain with focus on myeloarchitecture.^1^ In the first decade of the 20th century, Korbinian Brodmann—who was assistant of the Vogts in Berlin—published seminal papers about the cytoarchitecture of various brain regions, which he then integrated and expanded in his 1909 monograph that includes his famous cytoarchitectonic map with 43 areas (for an overview see Zilles^3^). While Brodmann's map is widely cited and dominates his legacy, Vogt's myeloarchitectonic approach was less well known for many years and is only currently experiencing something of a renaissance.^4,5^

At the turn of the 19th to the 20th century, new ideas and approaches emerged in different places and laboratories, changing the fields of neurology, psychiatry and neurosurgery and leading to progress in basic brain research. Oskar Vogt worked in the laboratory of Paul Flechsig in Leipzig, who studied the basis of myelinization during development, with Joseph Jules Dejerine in Paris, who published one of the most comprehensive textbooks on connectivity and anatomy and with Otto Binswanger in Jena, one of the founders of neuropsychiatry. Cécile Vogt studied in Pierre Marie's lab in Paris, a famous neurologist and neuropathologist, where she met Oskar, whom she married 1899.

The interest of the Vogts in the brain was driven by questions coming from neurology and psychiatry. Against this background, they developed their own ambitious research program and brought together a strong team of researchers and technicians in Berlin (Fig. 1). Moreover, the Vogts collaborated with Otfried Foerster, a distinguished neurologist and neurosurgeon, to explore the relationship of neurostimulation with the underlying brain anatomy. Such comparisons were facilitated by stimulation experiments in monkey brains, which were later studied histologically (Fig. 1).

Interestingly, the research programme of the Vogts combined different disciplines, including neuroanatomy, architectonics, histology, histopathology and electrophysiology, with neurochemistry and pharmacology, experimental and human genetics, physiology, psychology and phonetics. Accordingly, Oskar Vogt conceived and founded the Kaiser Wilhelm Institute for Brain Research in Berlin with such departments, where he was until 1937.

The development of this program was tightly linked with an increasing number of histological sections from brains of healthy human subjects, patients, non-human primates and other species. In total, the Vogts generated sections from about 1600 human brains and more than 100 species of non-human primates and other animals including rats, bats, deer, cats, ravens, dolphins and horses. Some sections result from cutting blocks of hemispheres or slabs of hemispheres, while others were processed as entire organs. Most of the sections have been stained either for cell bodies or myelin. To be able to process large sections, a new microtome was designed and manufactured. Embedding and staining techniques were improved, novel photographic techniques developed, and processes carefully monitored and documented. Altogether, the histological collection was stored on approximately 28 000 shelves in 70 collection cabinets, each with up to 400 shelves. Sometimes shelves were occupied by several layers of sections. These sections are well documented in protocols and patient folders. In addition, the Vogts collected about 11 000 glass plates, slides and photos, 20 000 reprints and various other objects.

The enormous quantity of histological sections is certainly much more than can be analysed in a single researcher's lifetime, and it would be interesting to know what the Vogts thought about this. The answer lies perhaps in the 12 000 sheets in more than 1200 bound volumes—the basis for the Vogt Archive—which also have been preserved.

After the Vogts’ death, the Collection with its various components was moved to Düsseldorf, Germany, where it is now hosted by the Cécile and Oskar Vogt Institute for Brain Research at the University Hospital Düsseldorf (https://www.uniklinik-duesseldorf.de/patienten-besucher/klinikeninstitutezentren/c-u-o-vogt-institut-fuer-hirnforschung/sammlungen-1). The Collection was maintained and expanded by the successors, Adolf Hopf (director 1960–88) and later Karl Zilles (until 2012). The author of this essay took over in 2013. Adolf Hopf and Karl Zilles also took care of the written legacy: the Vogt Archive was developed by Ursula Grell from the 1990s. It allowed her to respond to requests from researchers worldwide to use material from the archive. The resulting publications include books, monographs and journal articles that shed light on the Vogts, their collections and contemporary history from various perspectives (for an overview of the literature see the Supplementary material). However, the spatial conditions in which the collection and the archive have been stored, and their physical and non-digital presence do not allow the Vogt Collection to be made accessible to a broader scientific public.

The histological collection was studied rather little, except for one brain, which was used as a control or reference brain in several publications, for example, Mai *et al*.^5^ The patient records and protocols were stored in folders and were almost untouched since the 1950s. After the death of the two Vogts, several studies of the Vogt school were published (e.g. by Gerhard, Beheim-Schwarzbach and later Hopf; Supplementary material), which contributed detailed, albeit difficult to reproduce, descriptions of various brain regions. For a long time, more research was done on the collection than with the collection. As is often the case with anatomical collections, large parts were stored in basement rooms that were difficult to access. In this case, however, they were kept dry, clean and safe. Thus, they are in good shape and can now be ‘reactivated’. The conceptual, methodical, technical and informatics basis to handle such a large collection is available nowadays. Digitalization of the histological sections and documentations seems to be straight forward. But what are the arguments to start such a gigantic project? What are the historical and ethical preconditions? These must guide neuroscientific research, and they must be in place from the very beginning. An increase of scientific use is therefore only possible with deep historical research.

One argument is that in the past, histological collections have contributed many times to progress in brain research. For example, the Yakovlev collection was instrumental in better understanding brain development and pushed quantitative studies.^6^ Many sections are invaluable, and without the preservation of this collection there would be no access to them anymore. While this has been true for a long time, interesting new lines of research have also emerged, that shed new light on the historical sections of the Vogts and similar collections:

- A large number of brains have been processed in a systematic way: serial sections have been obtained, often stained in an alternating way for Nissl and myelin. This allows us to 3D-reconstruct the section series, and to create histological volumes. Single sections as well as histological volumes can be aligned to commonly used reference spaces such as the MNI space or the *BigBrain*^7^ as illustrated in Fig. 2. Such co-registration of sections to modern 3D template spaces links the information from the historical sections regarding cell and myelin distribution to a broad variety of multimodal data on function, connectivity, molecular architecture and all kinds of omics data in a multimodal atlas (e.g. https://www.ebrains.eu/): the analysis of the historical sections would benefit from a knowledge space of modern digital atlases that gives the sections a spatial and semantic context.
- Progress in optical and digital methods allows to extract fibre information from unstained sections and Nissl-stained sections.^8,9^ This opens new opportunities to extract multimodal information from histological sections beyond the original staining target. Such a perspective would significantly increase the impact of historical brain collections with their huge number of Nissl-stained sections from brains of different ages, medication and epidemiological background, spanning a more than 100-year history.
- In recent years, new genetic methods including next generation sequencing, shotgun sequencing and techniques for DNA repair and enrichment have revolutionized the examination of tissue, some of which is extremely old. In combination with other tools, e.g. organoids, this allowed conclusions to be made about differences between neurogenesis of modern humans and Neanderthals.^10^ It can be assumed that those methods will increasingly be incorporated into work with historical collections.
- The histological sections are closely linked to the documents of the Archive, the clinical records, photographs, drawings, publications based on the brain collection, correspondences and other parts of the Vogt Collection. The Vogt Collection therefore contains a wide range of different objects that are linked to each other in a complex way. It is vital to represent this complexity, and to be able to share it with a broader scientific community. This must be achieved by combining modern data science (e.g. metadata standards) with neuroscience, neurology, ethics and history. Finally, digitalization requires a significant data storage, and computing capacities for data sharing and processing, which only became available in recent years.^8^

**Figure 2:**
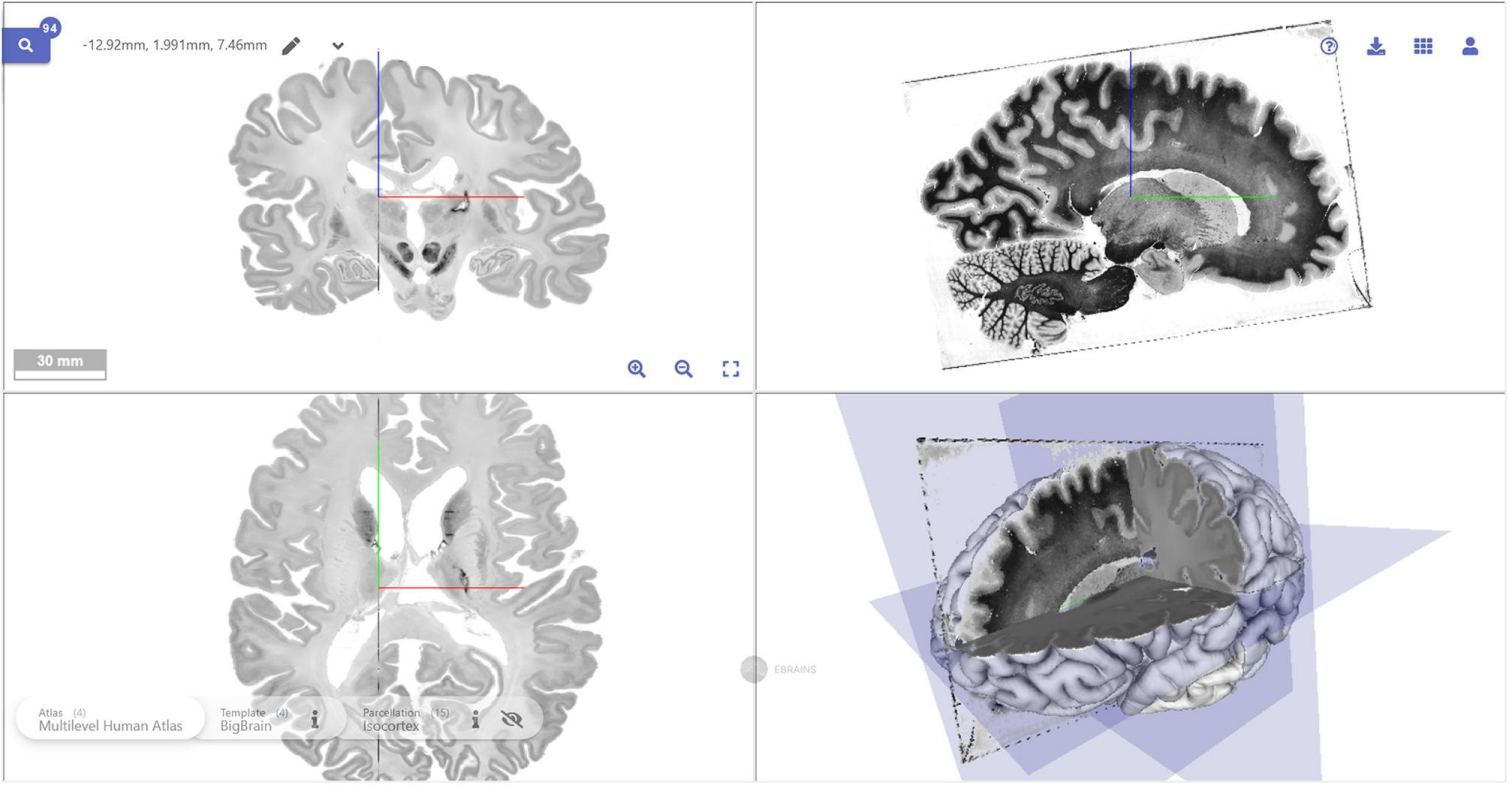
**Myeloarchitectonic section of the Vogt collection (from Fig. 1B) scanned and aligned to the *BigBrain* template**. The *BigBrain* is an anatomical model based on the 3D reconstruction of 7404 coronal histological sections.^7^ Its spatial resolution is 20 μm isotropic. It serves as a microscopic reference space in the multi-level human brain atlas of the EBRAINS research infrastructure (https://www.ebrains.eu/). The digitized myeloarchitectonic section has been aligned using an affine transformation into the *BigBrain* space. This illustrates the feasibility for aligning historical sections to modern, digital 3D atlases, and to make them available as FAIR data.

Such lines of research seem to align well with Vogt's vision of bringing together different disciplines, including genetics, surgery and pharmacology, through innovative methods, to improve our understanding of the pathogenesis of neurological diseases.

Although many of the Vogts’ views and research results have been overturned by new findings over the years, the Vogt Collection remains an invaluable part of our cultural heritage, offering a unique reflection of science and its history. It was assembled over a period of six decades, a period marked by major social upheavals. Importantly, this included the era of National Socialism. Although the Vogts were persecuted by the Nazis and were not involved in their euthanasia programmes, it has been known since 1988 at the latest that some of the existing sections were resulting from such programmes after the murder of patients and later were included in the collection through the Vogts’ personal relationships, e.g. with Bernhard Patzig [see Bogerts (1988) in the Supplementary material]. In response, my predecessor has decided to remove such sections from research. A project for in-depth investigation is now planned to address this issue.

When the six decades of the Vogt's research is evaluated in relation to today, new neuroscientific and ethical concepts have emerged, and changes in the organization of research itself can be observed. Whether for the history of neurology, psychiatry and psychology, for sociological, ethical and political aspects of 20th century sciences, or for research into the brain in all its facets, the Vogt Collection and Archive is a treasure trove for research in a wide range of areas from basic science to medicine, history and ethics. Building on the archive and the digitization of all histological sections and documents, this unique collection is now to be fully catalogued and made accessible to the broad scientific public in order to make it available as a source and inspiration for research.

## Supplementary Material

awaf365_Supplementary_Data

## Data Availability

This essay does not include any original data. However, access to use of the private Vogt Archive for scientific purposes is possible after submitting a user request. Please contact us at vogt-archiv@uni-duesseldorf.de. The histological collection is being moved to a new building currently and access is restricted.
