# Supplementary material for: The Vogt Collection: reactivating a treasure facilitating brain research, neurology and psychiatry
Source: Brain. 2025 Oct 1;149(1):2–5. doi: 10.1093/brain/awaf365 (PMC12782160; doi:10.1093/brain/awaf365)
Supplement: awaf365_Supplementary_Data [file awaf365_supplementary_data.pdf]

Supplement

1. Publications of Cécile and Oskar Vogt

Further information available at

https://www.uniklinik-duesseldorf.de/patienten-besucher/klinikeninstitutezentren/c-u-o-vogt-institut-fuer-hirnforschung/sammlungen-1

https://becker.wustl.edu/news/publications-cecile-and-oskar-vogt-and-letters-oskar-vogt-now-available/

1. Vogt, O. (1895). *Zur Kenntnis des Wesens und der psychologischen Bedeutung des Hypnotismus*: Johann Ambrosius Barth.
2. Vogt, O. (1897). *Die directe psychologische Experimentalmethode in hypnotischen Bewusstseinzuständen*: Johann Ambrosius Barth.
3. Vogt, O. (1897). *Die Zielvorstellung der Suggestion*: Johann Ambrosius.
4. Vogt, O. (1897). *Spontane Somnambulie in der Hypnose*: Johann Ambrosius Barth.
5. Vogt, O. (1897). Flechsig's Associationscentrenlehre, ihre Anhänger und Gegner. *Zeitschrift für Hypnotismus, 5*, 347-361.
6. Vogt, O. (1898). Sur la myélinisation de l'hémisphère cérébral du chat. *Compt. Rend. Soc. Biol. Paris, 50*, 54-56.
7. Vogt, O. (1899). Zur Methodik der ätiologischen Erforschung der Hysterie. *Zeitschrift für Hypnotismus, 8*, 65-83.
8. Vogt, O. (1899). Über die Natur der suggerirten Anästhesie. *Zeitschrift für Hypnotismus, 7*(6), 336-341.
9. Vogt, O. (1900). Ueber die Errichtung neurologischer Centralstationen. *Zeitschrift für Hypnotismus (Journal für Psychologie und Neurologie), 10*, 170-177.
10. Vogt, O. (1900). Sur la nécessité de fonder des instituts centraux pour l'anatomie du cerveau. *Rev. neurol. 8*, 723.
11. Vogt, O. (1900). *Résumé et conclusions du Rapport sur La Valeur de l'hypnotisme comme moyen d'investigation psychologique.* Paper presented at the: Deuxième congrès international de l'hypnotisme expérimental et thérapeutique, Paris.
12. Vogt, O. (1900). Die möglichen Formen seelischer Einwirkung in ihrer ärztlichen Bedeutung. Eine programmartige Übersicht. *Zeitschrift für Hypnotismus, 11*(6), 353-370.
13. Vogt, O. (1900). Kurze Bemerkungen zu den vorstehenden kritischen Bemerkungen Hirschlaff's. *Zeitschrift für Hypnotismus, 9*(4), 229-230.
14. Vogt, O. (1900). Zur Indication der Beschäftigungstherapie bei functionellen Nervenkranken. *Wiener klinischen Rundschau, 2 u. 3*.
15. Vogt, O. (1900). Valeur de l'étude de la myélinisation pour l'anatomie et la physiologie du cerveau. *Journal de Physiologie et de Pathologie gébérale, 4*, 525-548.
16. Vogt, C. (1901). Zur Hirnfaserungslehre. *Allg. Zeitschr. f. Psychiatrie. 58*.
17. Vogt, O. (1901). *Ueber centralisiertes hirnanatomisches Arbeiten.* Verhandlungen des Kongresses für Innere Medizin. In: Bergmann Wiesbaden.
18. Vogt, O. (1902). Zur Erweiterung unserer Zeitschrift. *Zeitschrift für Hypnotismus (Journal für Psychologie und Neurologie), 10*, 376-380.
19. Vogt, O. & Vogt, C. (1902). L'anatomie du cerveau et la psychologie. *CR IVe Congrès*.

20. Vogt, O. & Vogt, C. (1902). Zur Erforschung der Hirnfaserung.
21. Vogt, O. (1902). Psychologie, Neurophysiologie und Neuroanatomie. *Journal für Psychologie und Neurologie* 1(1): 1-3.
22. Vogt, O. (1902). Zur Erweiterung unserer Zeitschrift. *Zeitschrift für Hypnotismus (Journal für Psychologie und Neurologie), 10*, 376-380.
23. Vogt, O. (1902). Die möglichen Formen seelischer Einwirkung in ihrer ärztlichen Bedeutung. Eine programmässige Übersicht. *Journal für Psychologie und Neurologie, 1*(4), 146-160.
24. Vogt, O. (1902). Über den Einfluss einiger psychischer Zustände auf Kniephänomen und Muskeltonus. *Zeitschrift für Hypnotismus, 5*(4), 202-218.
25. Isenberg, D. & Vogt, O. (1902). Zur Kenntnis des Einflusses einiger psychischer Zustände auf die Athmung. *Zeitschrift für Hypnotismus, 10*(3), 131-158.
26. Vogt, O. (1903). Erklärung zum Fall Dippold. *DMW-Deutsche Medizinische Wochenschrift, 29*(43), 791-792.
27. Vogt, O. & Vogt, C. (1903). Zur anatomischen Gliederung des Cortex cerebri. *Journ. f. Psychol. u. Neurol. 2*(4), 160-180.
28. Vogt, C. & Vogt, O. (1904). *Die Markreifung des Kindergehirns während der ersten vier Lebensmonate und ihre methodologische Bedeutung; Forts. 1. Atlas; Teil 1. Tafel 1-124: Cécile und Oskar Vogt*: Fischer.
29. Vogt, O. (1904). Die hirnanatomische Abteilung des Berliner Neuro-Biologischen Universitäts-Laboratoriums mit besonderer Berücksichtigung ihrer bisherigen Resultate auf dem Gebiete der Reproduktionstechnik. *Anat. Anz, 27*(Suppl.), 79-83.
30. Vogt, O. (1905). Das Pantomikrotom des Neurobiologischen Laboratoriums. *Journ. f. Psychol. u. Neurol. 6*(3/4), 121-125.
31. Vogt, O. (1906). Der Wert der myelogenetischen Felder der Großhirnrinde. *Anat Anz (Jena), 29*, 273-287.
32. Vogt, C. (1907). Zur Kenntnis der electrisch erregbaren Hirnrinden-Gebiete bei den Saugetieren. *Journ. f. Psychol. u. Neurol. 8*, 277-456.
33. Vogt, C. (1909). La myéloarchitecture du thalamus du cercopithèque. *Journ. f. Psychol. u. Neurol. 12*, 285.
34. Vogt, O. (1910). Die myeloarchitektonische Felderung es menschlichen Stirnhirns. *Journ. f. Psychol. u. Neurol. 15*(4/5), 221-232.
35. Vogt, O. (1911). Die Myeloarchitektonik des Isocortex parietalis. *Journ. f. Psychol. u. Neurol. 18*, 379-390.
36. Vogt, O. & Vogt, O. (1911). *Nouvelle contribution à l'étude de la myéloarchitecture de l'écorce cérébrale.* Paper presented at the XX. Congres des médecins aliénistes et neurologistes de France, Brüssel.
37. Vogt, O. (1911). *La nouvelle division myéloarchitecturale de l'écorce cérébrale et ses rapports avec la physiologie et la psychologie*: Johann Ambrosius Barth.
38. Vogt, C. & Oppenheim, H. (1911). Wesen und Lokalisation der kongenitalen und infantilen Pseudobulbärparalyse. *J. f. Psychiat. u. Neurol, 18*, 293.
39. Vogt, C. (1911). Quelques considérations générales à propos du syndrome du corps strié. *J. f. Psychol. u. Neurol. 18*, 479-488.
40. Freund, C. S. & Vogt, C. (1911). Ein neuer Fall von Etat marbré des Corpus striatum. Un nouveau cas d'etat marbre du corps strie. *J. f. Psychol. u. Neurol. 18*, 489-500.
41. Vogt, O. (1911). Studien über das Artproblem. 2. *Mitteilung. Über das Variieren der Hummeln, 2*, 31-74.
42. Vogt, O. (1911). Studien über das Artproblem. Mitt. 1. Über das Variiren der Hummeln. T. 2. *Sitzungsberichte der Gesellschaft Naturforschender Freunde zu Berlin*, 31-74.

43. Vogt, O. (1912). Bedeutung, Ziele und Wege der Hirnforschung. *Nord Süd, 36*, 309-314.
44. Vogt, O. (1912). *Die neuen allgemeinen Feststellungen der Rindenarchitektonik.* Paper presented at the: Bericht über den Vten Kongress für experimentelle Psychologie, Leipzig.
45. Vogt, O. (1912). Über Forscher und Organisation der Forschung. *Nord und Süd. Eine deutsche Monatsschrift, 37*(459), 346-357.
46. Vogt, O. (1912). Bedeutung, Ziele und Wege der Hirnforschung. *Nord Süd, 36*, 309-314.
47. Vogt, O. (1913, 13 novembre 1913). *L'architecture et les localisations corticales.* Paper presented at the : Société de Neurologie de Paris, Paris.
48. Vogt, C. & Vogt, O. (1915). Über einen angeblichen Fasciculus corporis callosi cruciatus. *Journ. f. Psychol. u. Neurol. 21*(3/4), 154-158.
49. Vogt, C. & Vogt, O. (1919). *Allgemeine Ergebnisse unserer Hirnforschung* (Vol. 25): JA Barth.
50. Vogt, C. & Vogt, O. (1919). Erster Versuch einer pathologisch-anatomischen Einteilung striärer Motilitätsstörungen nebst Bemerkungen über seine allgemeine wissenschaftliche Bedeutung. *J. f. Psychol. u. Neurol. 24*(1).
51. Vogt, C. Vogt, O. Vogt, O. Neurologist, G. & Vogt, O. (1919). *Zur Kenntnis der pathologischen Veränderungen des Striatum und des Pallidum und zur Pathophysiologie der dabei auftretenden Krankheitserscheinungen*.
52. Vogt, O. (1919). Die Diplomatie als angewandte Psychologie. *Nord und Süd, 168*, 123-126.
53. Vogt, C. & Vogt, O. (1919). Wissenschaftliche Forderungen an den modernen Staat. *Nord und Süd, 168*, 245-250.
54. Vogt, O. (1920). Das Kaiser-Wilhelm-Institut für Hirnforschung. *Journ. f. Psychol. u. Neurol.* 219.
55. Vogt, O. (1920). Über zwei wichtige Aufgaben der Erforschung des Hundes und der Brieftaube. . *Journ. f. Psychol. u. Neurol. 25*, 219.
56. Vogt, C. (1921). Einige Ergebnisse unserer Neurosenforschung. *Naturwissenschaften, 9*(18), 346-350.
57. Vogt, C. & Vogt, O. (1921). Die Bedeutung der topistischen und pathologischanatomischen Erforschung des Nervensystems für die Lehre von seinen Erkrankungen. In *Festschrift der Kaiser Wilhelm Gesellschaft zur Förderung der Wissenschaften zu ihrem Zehnjährigen Jubiläum Dargebracht von ihren Instituten* (pp. 218-223): Springer.
58. Vogt, O. (1921). Ergebnisse der Analyse gewisser Merkmale einiger Insektengattungen. *Naturwissenschaften, 9*(18), 350-353.
59. Vogt, C. (1921). *Die Pathoarchitektonik als besonderer Zweig der pathologischen Anatomie des Zentralnervensystems.* Stockholm.
60. Vogt, O. & Vogt, C. (1922). *Erkrankungen der Grosshirnrinde im Lichte der Topistik, Pathoklise und Pathoarchitektonik, von Cécile und Oskar Vogt*: JA Barth.
61. Vogt, O. (1923). Furchenbildung und architektonische Rindenfelderung. *Journ. f. Psychol. u. Neurol. 29*, 438-439.
62. Bárány, R. Vogt, C. & Vogt, O. (1923). Zur reizphysiologischen Analyse der kortikalen Augenbewegungen. *Journ. f. Psychol. u. Neurol. 30*, 87-121.
63. Vogt, C. & Vogt, O. (1923). De las alteraciones patoarquitectónicas causales de las enfermedades mentales, como casos extremos de la variación normal en las especies. *Revista médica de Hamburgo, 4*(5), 137-144.
64. Vogt, O. (1925). Ein weiterer Beitrag zur elektiven Natur der pathoarchitektonischen Veränderungen der Großhirnrinde. *Mémoires publiés à l'occasion du jubilé du Prof. Rossolimo*.
65. Vogt, C. (1925). Sur l'état marbré du striatum. *Journ. f. Psychol. u. Neurol. 31*, 250-260.
66. Vogt, O. (1925). Der Begriff der Pathoklise. *Journ. f. Psychol. u. Neurol, 31*(5), 245-260.
67. Vogt, C. V. Oskar. (1926). Die vergleichend-architektonische und die vergleichend-

reizphysiologische Felderung der Großhirnrinde unter besonderer Berücksichtigung der menschlichen. *Naturwissenschaften, 14*, 1190–1194 doi:https://doi.org/10.1007/BF01451766
68. Vogt, C. (1926). Die topistisch-pathoarchitektonische Forschung. *Zeitschrift für die gesamte Neurologie und Psychiatrie, 100*, 63.
69. Vogt, C. (1926). Topistik und psychiatrische Klassifikation. *Zeitschrift für die gesamte Neurologie und Psychiatrie, 101*(1), 798-804.
70. Vogt, C. & Vogt, O. (1926). Die nosologische Stellung des Status marmoratus des Striatum. *Psychiat. Neurol. Wschr. 28*, 85-87.
71. Timoféeff-Ressovsky, N. & Vogt, O. (1926). Über idiosomatische Variationsgruppen und ihre Bedeutung für die Klassifikation der Krankheiten. *Naturwissenschaften, 14*(50), 1188-1190.
72. Vogt, O. (1927). Architektonik der menschlichen Hirnrinde. *Allg Z Psychiatr, 86*, 247-274.
73. Vogt, O. & Fersman, A. (1927). Die Russische Forscherwoche in Berlin. *Osteuropa, 2*(8/9), 459-465.
74. Vogt, C. & Vogt, O. (1928). Die Grundlagen und die Teildisziplinen der mikroskopischen Anatomie des Zentralnervensystems. In *Nervensystem: Erster Teil Nervengewebe das Peripherische Nervensystem das Zentralnervensystem* (pp. 448-477): Springer.
75. Vogt, C. & Vogt, O. (1928). *Zur psychiatrischen Würdigung der Antonschen Entdeckung und Wertung des Status marmotus striati*: Verlag von Johann Ambrosius Barth.
76. Vogt, O. (1929). Bericht über die Arbeiten des Moskauer Staatsinstituts für Hirnforschung. *J. Psychol. Neurol, 40*, 108-118.
77. Vogt, C. & Vogt, O. (1929). Hirnforschung und Genetik. *Journ. f. Psychol. u. Neurol. 39*, 438-446.
78. Vogt, O. (1929). Bericht über die Arbeiten des Moskauer Staatsinstituts für Hirnforschung. *Journ. f. Psychol. u. Neurol. 40*, 108-118.
79. Vogt, C. & Vogt, O. (1929). *Über die Neuheit und den Wert des Pathoklisenbegriffes*: Verlag von Johann Ambrosius Barth.
80. Vogt, O. & Zarapkin, S. R. (1929). *Über dysnomische Variabilität und ihre nosologische Bedeutung*: Verlag von Johann Ambrosius Barth.
81. Vogt, O. (1929). Bericht über die Arbeiten des Moskauer Staatsinstituts für Hirnforschung. *Journ. f. Psychol. u. Neurol. 40*, 108-118.
82. Vogt, O. & Vogt, C. (1930). Cytoarchitektonik und Hirnlokalisation. *Psychiatrisch-Neurologische Wochenschrift, 32*, 127-128.
83. Vogt, C. & Vogt, O. (1930). Weitere biologische Beleuchtungen des Problems der Klassifikation der Erkrankungen des Nervensystems. *Zeitschrift für die gesamte Neurologie und Psychiatrie, 128*(1), 557-575.
84. Vogt, C. & Vogt, O. (1931). Über funktionelle und genetische Harmonieen. *European Neurology, 80*(1-2), 115-119.
85. Vogt, O. (1931). Warum treiben wir Hirnforschung? *Forschungen und Fortschritte, 7*, 309.
86. Vogt, O. (1932). Neurology and eugenics: the role of experimental genetics in their development. *The Eugenics Review, 24*(1), 15.
87. Vogt, C. (1933). Warum stellen wir die Hirnanatomie in den Mittelpunkt unserer Forschung? *Naturwissenschaften, 21*(21), 408-410.
88. Vogt, O. (1933). Über biologische Harmonien. *Naturwissenschaften, 21*(21), 406-408.
89. Vogt, C. & Vogt, O. (1935). Zur spezifischen Variabilität unserer Orange. *Naturwissenschaften, 23*(26), 496-499.
90. Vogt, C. & Vogt, O. (1937). Sitz und Wesen der Krankheiten im Lichte der topistischen Hirnforschung und des Variierens der Tiere. *Journ. f. Psychol. u. Neurol. 47,* 238-457.
91. Vogt, C. & Vogt, O. (1940). *Das formative Sonderverhalten des einzelnen Griseum cerebrale*: Johann Ambrosius Barth.

92. Vogt, C. & Vogt, O. (1941). Thalamusstudien I-III. *J. Psychol. Neurol. Lpz, 50*, 31-154.
93. Vogt, O. (1941). *Der heutige Stand der cerebralen Organologie und die zukünftige Hirnforschung: Eine Huldigung für Theodor Meynert anlässlich der 50. Wiederkehr seines Todestages*: Gustav Fischer.
94. Vogt, C. & Vogt, O. (1942). *Morphologische Gestaltungen unter normalen und pathogenen Bedingungen: Ein hirnanatomischer Beitrag zu ihrer Kenntnis*: Barth.
95. Vogt, C. & Vogt, O. (1944). Tätigkeit verzögert das Altern. *Kurznachrichten. Beiblätter zu "Forschungen und Fortschritte". 7*, 26-27.
96. Vogt, C. & Vogt, O. (1946). Eine neurohistologische Beleuchtung der Nucleolusfunktion. *Biol. Zbl. 65*, 61-69.
97. Vogt, C. & Vogt, O. (1947). Über Wesen und Ursache des Alterns der Hirnzellen. *Forschungen Fortschr, 21*(23), 4-6.
98. Vogt, C. & Vogt, O. (1947). Lebensgeschichte, Funktion und Tätigkeitsregulierung des Nucleolus. *Ärztl. Forsch. 1*(8), 43.
99. Vogt, C. & Vogt, O. (1947). Wesen und orthologische Bedeutung der pathologischen Erscheinungen. *Nervenarzt, 18*, 97-103.
100. Vogt, O. (1947). Ethnos, ein neuer Begriff der Populations-Taxionomie. *Naturwissenschaften, 34*(2), 45-52.
101. Vogt, C. & Vogt, O. (1947). Ätiologie und Erkrankungsbild: I. Mitteilung. *Klinische Wochenschrift, 24*(39), 609-621.
102. Vogt, C. & Vogt, O. (1948). Über anatomische Substrate. Bemerkungen zu pathoanatomischen Befunden bei Schizophrenen. *Ärztl Forsch, 3*, 1-7.
103. Vogt, O. (1948). Der Erkenntniswert der heutigen Hirnanatomie. Zum Hundertsten Geburtstag August Forels. *Schweizerischen Medizinischen Wochenzeitschrift, 78*(34), 837.
104. Vogt, C. & Vogt, O. (1949). Biologische Grundanschauungen. Zugleich eine Basis für die Kritik anatomischer Hirnveränderungen bei Schizophrenen. *Ärztl. Forsch. 3*, 121.
105. Vogt, C. & Vogt, O. (1949). *Differente histologische Prozesse im Nucleus caudatus und im Putamen bei bilateraler progressiver Chorea.* (Nach einer Demonstration im Neustädter Hirnforschungs-Institut am 23. 9. 1949 im Selbstverlag.).
106. Vogt, C. & Vogt, O. (1950). Wie weit lassen sich schon heute bei Funktionsanomalien des Gehirns anatomische Besonderheiten nachweisen? *Der Nervenarzt, 21*(8), 337-339.
107. Vogt, O. (1951). Die anatomische Vertiefung der menschlichen Hirnlokalisation. *Klinische Wochenschrift, 29*(7), 111-125.
108. Vogt, C. & Vogt, O. (1951). Importance of neuroanatomy in the field of neuropathology. *Neurology, 1*(5), 205-205.
109. Vogt O. (1952). Korbinian Brodmann (1868-1918). In: Kolle K, editor. Große Nervenärzte. Vol. 2. Stuttgart: Thieme; 1952. p. 40–4.
110. Vogt, C. & Vogt, O. (1952). Résultats de l'étude anatomique de la schizophrenie et d'autres psychoses dites fontionelles faite à I'institut du cerveau de Neustadt, Schwarzwald. *Proc 1st Int Congr Neuropath, 1*, 515-532.
111. Vogt, C. & Vogt, O. (1952). *Alterations anatomiques de la schizophrenie et d'autres psychoses dites fonctionelles.* Paper presented at the Proceedings of the first international congress of neuropathology.
112. Vogt, C. (1952). *Die Bedeutung der pathologischen Anatomie für eine ätiologische Klassifikation der Gehirnkrankheiten.* Paper presented at the Klinische Wochenschrift.
113. Vogt, O. (1952). Precipitating and modifying agents in chorea. *The Journal of nervous and mental disease, 116*(6), 601-607.
114. Vogt, O. (1952). *Proposition de fonder une organisation internationale pour l'etude de l'anatomie*

pathologique de la schizophrenie et d'autres psychoses dites fonctionnelles. Paper presented at the Proceedings of the First International Congress on Neuropathology.

115. Vogt, C. & Vogt, O. (1953). *Gestaltung der topistischen Hirnforschung und ihre Förderung durch den Hirnbau und seine Anomalien*: Akademie-Verlag.
116. Vogt, O. (1953). Alter und Untätigkeit. *Die Gegenwart, 186*.
117. Vogt, C. & Vogt, O. (1953). Vorbemerkungen zu einer ätiologischen Klassifikation der Schizophrenie und anderer „funktioneller "Psychosen. *Psychiatrie, Neurologie und medizinische Psychologie, 5*(1/2), 4-8.
118. Vogt, C. & Vogt, O. (1954). Gestaltung der topistischen Hirnforschung und ihre Förderung durch den Hirnbau und seine Anomalien. *Journal für Hirnforschung,* Heft 1/2.
119. Vogt, C. & Vogt, O. (1956). Weitere Ausführungen zum Arbeitsprogramm des Hirnforschungsinstitutes in Neustadt/Schwarzwald. *J Hirnforsch, 2*, 403-427.

2. About the Vogts and the Vogt collection

This list includes a selection of journal articles, books, and book chapters about the Vogts, their research and brain collection.

1. Hansson, N., Fangerau, H., de Sio F., Grell, U., Amunts, K., Pioneers of modern brain research—Cécile and Oskar Vogt and the Nobel Prize. Frontiers in Neuroanatomy, 2025. Volume 19 - 2025. https://doi.org/10.3389/fnana.2025.1679993
2. Kasper, B. S. (2025). Cécile & Oskar Vogt: Leben, Werk und ihr Beitrag zur Epileptologie. *Clinical Epileptology*.38, 122–131
3. Winterhalder, M. (2023). *Gehirn und menschliche Natur. Die neuropsychologischen Forschungen Kurt Goldsteins sowie Cécile und Oskar Vogts, 1895-1936*: transcript Verlag.
4. Kofler-Bettschart, B. (2022). *Cécile Vogt: Pionierin der Hirnforschung* (1. Auflage): Carl Ueberreuter Verlag GmbH.
5. Hildebrandt, S. (2020). Anatomy in Nazi Germany: The Use of Victims' Bodies in Academia and Present-Day Legacies: (Video presentation can be accessed from the HTML). *Journal of Biocommunication*. 45(1). https:/doi.org/10.5210/jbc.v45i1.10848
6. Martin, M. Karenberg, A. & Fangerau, H. (2020). Neurowissenschaftler am Kaiser-Wilhelm-Institut für Hirnforschung im „Dritten Reich ": Oskar Vogt–Hugo Spatz–Wilhelm Tönnis. *Der Nervenarzt, 91*(Suppl 1), 89-99.
7. Akkermans, R. (2018). Cecile Vogt. *Lancet Neurol, 17*(10), 846. doi:10.1016/S1474-4422(18)30002-4
8. Rubin, R. P. (2017). The Vogt family: Creators of diverse paths for women in biological research. *J Med Biogr, 25*(4), 252-260. doi:10.1177/0967772017731300
9. Marazia, C. & Fangerau, H. (2018). Chapter 7 - Imagining the brain as a book: Oskar and Cécile Vogt's "library of brains". In C. Ambrosio & W. MacLehose (Eds.), *Progress in Brain Research* (Vol. 243, pp. 181-203): Elsevier.
10. Koppitz, U. Labisch, A. & Schwarzburger, M.-I. (2017). Gelehrtenbibliotheken. Auf den Spuren der Neurowissenschaftler Oskar Vogt und John C. Eccles. In I. Siebert (Ed.), *"Das Paradeis fanden wir ..." Streifzüge durch die Bücherwelten der ULB Düsseldorf* (pp. 135-152). Frankfurt am Main: Vittorio Klostermann.
11. Breathnach, C. S. & Moynihan, J. B. (2013). First ladies in laying the foundation of neuroendocrinology. *Ir J Med Sci, 182*(1), 143-147. doi:10.1007/s11845-012-0830-9
12. Satzinger, H. (2011). Außenseiter: Cécile und Oskar Vogts Hirnforschung um 1900. in: Bleker, Johanna; Hulverscheidt, M. & Lenning, P. (Hrsg.): Visiten. Berliner Impulse zur Entwicklung der modernen Medizin (Berlin: Kulturverlag Kadmos, 2011), 179-195. DOI: https://doi.org/10.25595/241.

13. Zilles, K. (2011). *Oskar Vogt - ein Wegbereiter der Hirnforschung*, in *Life Sciences - Die Neukonstruktion des Menschen?*, A. Bamm, Editor. Profil Verlag: München-Wien. p. 35-76.
14. Judaš, M. & Cepanec, M. (2010). Oskar Vogt: The first myeloarchitectonic map of the human frontal cortex. *Translational Neuroscience, 1*(1), 72-94.
15. Wolff, H.-P. (2009). *Cécile und Oskar Vogt: eine illustrierte Biographie*: IFF-Inst. für Technik-und Wissenschaftsforschung.
16. Hagner, M. (2004). *Geniale Gehirne zur Geschichte der Elitegehirnforschung* (2. Aufl.).
17. v. Stuckrad-Barre, S. & Danek, A. (2004). Oskar Vogt (1870–1959). *Der Nervenarzt, 75*(10), 1038-1041. doi:10.1007/s00115-004-1772-x
18. Klatzo, I. (2003). Cecile & Oskar Vogt: the significance of their contributions in modern neuroscience. *Acta Neurochir Suppl, 86*, 29-32. doi:10.1007/978-3-7091-0651-8_6
19. Satzinger, H. (2003). Illnesses as racial concepts: political and scientific dimensions of a biomedical research program by Cecile and Oskar Vogt between Tiflis and Berlin (1919-1939). *Medizinhist J, 37*(3-4), 301-350. Retrieved from https://www.ncbi.nlm.nih.gov/pubmed/12703266
20. Haas, L. F. (2002). Cecile Vogt (1875-1962). *J Neurol Neurosurg Psychiatry, 73*(3), 315. doi:10.1136/jnnp.73.3.315
21. Hagner, M. (2002). Das Genie und sein Gehirn. In *Jahrbuch des Collegium Helveticum der ETH Zürich 2001* (pp. 187-211): vdf.
22. Klatzo, I. (2002). *Cécile and Oskar Vogt: The visionaries of modern neuroscience.* Springer Science & Business Media.
23. Reindl, J. (2002). Believers in an Age of Heresy? Oskar Vogt, Nikolai Timoféeff-Ressovsky and Julius Hallervorden at the Kaiser Wilhelm Institute for Brain Research. *German Historical Perspectives, 13*, 211-242.
24. Schmuhl, H.-W. (2002). Hirnforschung und Krankenmord. Das Kaiser-Wilhelm-Institut für Hirnforschung 1937-1945. *Vierteljahrshefte für Zeitgeschichte*, *50*(4), 559-609.
25. Holdorff, B. & Winau, R. (2001). *Geschichte der Neurologie in Berlin*: Walter de Gruyter.
26. Bentivoglio, M. (1998). Cortical structure and mental skills: Oskar Vogt and the legacy of Lenin's brain. *Brain Research Bulletin, 47*(4), 291-296.
27. Satzinger, H. (1998). *Die Geschichte der genetisch orientierten Hirnforschung von Cécile und Oskar Vogt (1875 - 1962, 1870 - 1959) in der Zeit von 1895 bis ca. 1927*: Deutscher Apotheker Verlag Stuttgart.
28. Satzinger, H. (1998). Weiblichkeit und Wissenschaft. Das Beispiel der Hirnforscherin Cecile Vogt (1875-1962). In: Matthiesen Verlag.
29. Grell, U. (1997). On the history of the C. and O. Vogt Institute for brain research. *Folia Anatomica, 25*(1), 17-24.
30. Mai, J. K., Lensing-Höhn, S., Ende, A. A., & Sofroniew, M. V. (1997). Developmental organization of neurophysin neurons in the human brain. *Journal of Comparative Neurology*, 385(3), 477-489.
31. Satzinger, H. (1996). Das Gehirn, die Frau und ein Unterschied in den Neurowissenschaften des 20. Jahrhunderts: Cécile Vogt (1875-1962). In C. R. Meinel, M (Ed.), *Geschlechterverhältnisse in Medizin, Naturwissenschaft und Technik* (pp. 75-82). Bassum, Stuttgart: Verlag für Geschichte der Naturwissenschaften und der Technik.
32. Landtblom, A. M. (1995). Early athetosis disclosed the function of the corpus striatum. Cecile Vogt described the syndrome. *Lakartidningen, 92*(49), 4691-4694. Retrieved from https://www.ncbi.nlm.nih.gov/pubmed/8531531
33. Fix M. (1994). Leben und Werk des Gehirnanatomen Korbinian Brodmann (1868-1918). Inaugural Dissertation, Tübingen: Eberhard-Karls-Universität Tübingen; 1994.
34. Kreutzberg, G. W. Klatzo, I. & Kleihues, P. (1992). Oskar and Cecile Vogt, Lenin's brain and the bumble-bees of the Black Forest. *Brain Pathology, 2*(4), 363-364.

35. Bogerts, B. (1990). Ueber die Hirne der Vogt-Sammlung [The brains of the Vogt collection]. *Nervenarzt, 61*(5), 315-316.
36. Kirsche, W. (1986). *Oskar Vogt 1870–1959: Leben und Werk und dessen Beziehung zur Hirnforschung der Gegenwart. Ein Beitrag zur 25. Wiederkehr seines Todestages*: De Gruyter.
37. Heintel, H. (1984). Ein unbekanntes Portrat des Hirnforschers Oskar Vogt (1870-1959). *Medizinhist J, 19*(3), 277-279. Retrieved from https://www.ncbi.nlm.nih.gov/pubmed/11611607
38. Honti, J. (1977). Husband-wife team Oscar and Cecile Vogt, scientific researchers of Lenin's brain. *Orv Hetil, 118*(14), 816-817. https://www.ncbi.nlm.nih.gov/pubmed/322021
39. Richter, J. (1976). Oskar Vogt and the founding of the Berlin Kaiser Wilhelm Institute for Brain Research under conditions of imperialistic scientific policy. *Psychiatr Neurol Med Psychol (Leipz), 28*(8), 449-457. Retrieved from https://www.ncbi.nlm.nih.gov/pubmed/794890
40. Richter, J. (1976). Oskar Vogt, founder of the Moscow State Institute for Brain Research. History of German-Soviet scientific relations in the field of neurology. *Psychiatr Neurol Med Psychol (Leipz), 28*(7), 385-395. Retrieved from https://www.ncbi.nlm.nih.gov/pubmed/785513
41. Kirsche, W. (1975). In memory of Cecile Vogt at the occasion of the 100tth anniversary of her birthday. *J Hirnforsch, 16*(1), 1-2. Retrieved from https://www.ncbi.nlm.nih.gov/pubmed/1102595
42. Schulze, H. A. (1971). Von der topistischen Hirnforschung zur dynamischen Lokalisationslehre: Zur 100. Wiederkehr des Geburtstages von Oskar Vogt am 6. April 1970. *Psychiatrie, Neurologie und medizinische Psychologie*, 4-7.
43. Hopf, A. (1970). Oskar Vogt. 100th anniversary of his birthday. *J Hirnforsch, 12*(1), 1-10. Retrieved from https://www.ncbi.nlm.nih.gov/pubmed/4925411
44. Rabl, R. (1970). Oskar Vogt, Gründer des Staatsinstituts für Hirnforschung in Moskau. *Die Waage, 9*, 65-73.
45. Sarkisov, S. A. Stankevich, I. A. Preobrazhenskaia, N. S. & Poliakov, G. I. (1970). Oskar Vogt (on the 100th anniversary of his birth). *Zh Nevropatol Psikhiatr Im S Korsakova, 70*(5), 763-764. Retrieved from https://www.ncbi.nlm.nih.gov/pubmed/4916172
46. Schulze, H. (1963). Die Begründung des wissenschaftlichen „Hypnotismus "in Deutschland durch Oskar Vogt. *Psychiatrie, Neurologie und medizinische Psychologie*, 189-192.
47. Hopf, A. (1962). In memoriam: Cecile VOGT. *J Hirnforsch, 5*, 245-248. Retrieved from https://www.ncbi.nlm.nih.gov/pubmed/13961661
48. Treff, W. M., & Hempel, K. J. (1962). Korrelation zwischen der Pathologie der Nervenzellen und den Zellveränderungen bei Schizophrenie. *J. Hirnforsch,* 5, 39-58.
49. Haymaker, W. (1961). Oskar VOGT, April 6, 1870-July 31, 1959. *Arch Neurol, 4*, 675-684. doi:10.1001/archneur.1961.00450120089010
50. Minkowski, M. (1961). Professor Oskar VOGT (1870-1959). *Schweiz Arch Neurol Neurochir Psychiatr, 87*, 305-314. Retrieved from https://www.ncbi.nlm.nih.gov/pubmed/13771099
51. Bruetsch, W. L. (1960). In memoriam Oskar Vogt, M.D. 1870-1959. *Am J Psychiatry, 116*, 958-960. doi:10.1176/ajp.116.10.958
52. Kern, A. (1960). In memoriam: Oskar VOGT. *Dtsch Gesundheitsw, 15*, 1545-1546. Retrieved from https://www.ncbi.nlm.nih.gov/pubmed/13752623
53. Kleist, K. (1960). In commemoration of Oskar VOGT. *Nervenarzt, 31*, 337-340. Retrieved from https://www.ncbi.nlm.nih.gov/pubmed/13756737
54. Silveira, A. (1960). In memoriam: Oskar Vogt. *Arq Neuropsiquiatr, 18*, 99-100. Retrieved from https://www.ncbi.nlm.nih.gov/pubmed/14446728
55. Wünscher, W. (1959). In memoriam Oskar Vogt. *Psychiatrie, Neurologie und medizinische Psychologie*, 289-290.
56. Hopf, A. (1959). In memoriam Oskar Vogt. *Ärztl Forsch, 13*, I/477-478. Retrieved from https://www.ncbi.nlm.nih.gov/pubmed/13855933

57. Kirsche, W. (1958). Das Institut für Hirnforschung und allgemeine Biologie in Neustadt im Schwarzwald: Bericht über einen Studienaufenthalt, einschließlich Perspektiven über die Stellung der Morphologie in der künftigen Hirnforschung. *Psychiatrie, Neurologie und medizinische Psychologie, 10*(12), 359-363.
58. Wahren, W. (1956a). Anatomische Untersuchungen am menschlichen Corpus geniculatum laterale. *J Hirnforsch,* 2, 78-93.
59. Wahren, W. (1956b). Kernuntersuchungen an der Makroglia. *J. Hirnforschung,* 2, 440-451.
60. Wünscher, W. (1956). Cytoarchitektonik und Involution einiger Hirnstammkerne mit vegetativen Funktionen und der oberen Olive. *Journal für Hirnforschung,* 2, 354-390.
61. Hassler, R. (1955). To Dr. Cecile Vogt on her 80th birthday. *Nervenarzt, 26*(10), 444-446. Retrieved from https://www.ncbi.nlm.nih.gov/pubmed/13297063.
62. Balthasar, K. (1954). Lebensgeschichte der vier größten Pyramidenzellarten in der V. Schicht der menschlichen Area gigantopyramidalis. *De Gruyter,* 281-325.
63. Haymaker, W. (1951). Cecile and Oskar Vogt, on the occasion of her 75th and his 80th birthday. *Neurology, 1*(3), 179-204. doi:10.1212/wnl.1.5.179
64. Schultz, J. H. (1951). Oskar Vogt in the history of German medical psychology. *Nervenarzt, 22*(2), 41-42. Retrieved from https://www.ncbi.nlm.nih.gov/pubmed/14815678
65. Olszewski, J. (1950). Cecile and Oskar Vogt. *AMA Arch Neurol Psychiatry, 64*(6), 812-822. doi:10.1001/archneurpsyc.1950.02310300059005
66. Kleist, K. (1950). Oskar Vogt, 80th anniversary, Cecile Vogt, 75th anniversary. *Arch Psychiatr Nervenkr Z Gesamte Neurol Psychiatr, 185*(6-7), 619-623. doi:10.1007/BF00935512
67. Pfeifer, R. A. (1950). In commemoration of the seventy-fifth birthday of Cecile Vogt and eightieth of Oskar Vogt. *Dtsch Gesundheitsw, 5*(24), 743-744. Retrieved from https://www.ncbi.nlm.nih.gov/pubmed/15427474
68. Hassler, T. (1950). [Jubilee of Oscar and Cecile Vogt, pioneers in encephalologic research]. *Dtsch Med Wochenschr, 75*(16), 556-557. Retrieved from https://www.ncbi.nlm.nih.gov/pubmed/15421136
69. Saldun De Rodrigues, M. L. & Pisciotane, V. (1947). Cecile Vogt's disease; double congenital athetosis. *Arch Pediatr Urug, 18*(12), 629-636. Retrieved from https://www.ncbi.nlm.nih.gov/pubmed/18905762

3. Neuroscientific publications connected to the Vogt Collection

This list includes publications that have used histological sections and/or their images of the Vogt collection to address a neuroscientific topic. Many of the early papers of the Vogt school, including those of Rose, Hopf, Gerhard or Beheim-Schwarzbach address the mapping of various brain regions. Korbinian Brodmann has published a series of papers on cytoarchitecture, before his monography was published 1909.

1. Zachlod D., Amunts K. (2025) Brodmann's map. *Oxford Research Encyclopedia of Neuroscience*. DOI:10.1093/acrefore/9780190264086.013.528
2. Kasper, B. S. Hebold, M. Stockmann, J.P. (2025). The first detailed study and illustration of Ammon's horn sclerosis in epilepsy and the men behind it: Emil Bratz (1868–1934) and Otto Hebold (1856–1945). *Clinical Epileptology*. doi: https://doi.org/10.1007/s10309-025-00748-2
3. Stacho, M. Hausler, A. N. Brandstetter, A. Iannilli, F. Mohlberg, H. Schiffer, C. . . . Amunts, K. (2024). Phylogenetic reduction of the magnocellular red nucleus in primates and inter-subject variability in humans. *Front Neuroanat, 18*, 1331305. doi:10.3389/fnana.2024.1331305
4. Ströckens, F. & Amunts, K. (2024). Human telencephalization. In N. Diederich, M. Brüne, K. Amunts, & C. Goetz (Eds.), *Evolutionary Roots of Human Brain Diseases.* (pp. 9-37). Oxford: Oxford University Press.

5. Nieuwenhuys, R. and M.F. Glasser (2024) A comparison of two maps of the human neocortex: the multimodal MRI-based parcellation of Glasser et al. (2016a), and the myeloarchitectonic parcellation of Nieuwenhuys and Broere (2023), as a first step toward a unified, canonical map. *Brain Struct Funct*, 229(9): p. 2509-2521.
6. Mai, J. K. & Majtanik, M. (2023). Myeloarchitectonic maps of the human cerebral cortex registered to surface and sections of a standard atlas brain. *Transl Neurosci, 14*(1), 20220325. doi:10.1515/tnsci-2022-0325
7. Nieuwenhuys, R. & Broere, C. A. J. (2023). A new 3D myeloarchitectonic map of the human neocortex based on data from the Vogt-Vogt school. *Brain Struct Funct, 228*(6), 1549-1559. doi:10.1007/s00429-023-02671-6
8. Axer, M. & Amunts, K. (2022). Scale matters: The nested human connectome. *Science, 378*(6619), 500-504. doi:10.1126/science.abq2599
9. Amunts K. (2021) Brodmann areas. *Encyclopedia of Evolutionary Psychological Science*, pp. 821-824: Springer International Publishing
10. Nieuwenhuys, R. & Broere, C. A. J. (2020). A detailed comparison of the cytoarchitectonic and myeloarchitectonic maps of the human neocortex produced by the Vogt-Vogt school. *Brain Struct Funct, 225*(9), 2717-2733. doi:10.1007/s00429-020-02150-2
11. Zilles, K. (2018). *Brodmann: a pioneer of human brain mapping—his impact on concepts of cortical organization. Brain*. 141(11): 3262-3278.
12. Amunts, K. (2018) Brodmann Areas: 1-3. *Encyclopedia of Evolutionary Psychological Science*, Cham: Springer International Publishing.
13. Amunts K, Zilles K. (2015). Architectonic mapping of the human brain beyond Brodmann. *Neuron* 2015; 88: 1086–107.
14. Mai, J.K., M. Majtanik, and G. Paxinos, Atlas of the human brain. 4th ed. 2015: Academic Press.
15. Nieuwenhuys, R., Broere, C. A., & Cerliani, L. (2015). A new myeloarchitectonic map of the human neocortex based on data from the Vogt-Vogt school. *Brain Struct Funct*, 220(5), 2551-2573. doi:10.1007/s00429-014-0806-9
16. Zilles K, Palomero-Gallagher N, Amunts K (2015) Myeloarchitecture and maps of the cerebral cortex. In Toga AW (Ed.), *Brain Mapping: An Encyclopedic Reference*: 137-156
17. Nieuwenhuys, R. (2013). The myeloarchitectonic studies on the human cerebral cortex of the Vogt-Vogt school, and their significance for the interpretation of functional neuroimaging data. *Brain Struct Funct*, 218(2), 303-352. doi:10.1007/s00429-012-0460-z
18. Judas M, Cepanec M, Sedmak G. Brodmann’s map of the human cerebral cortex – or Brodmann’s maps. *Transl Neurosci* 2012; 3: 67–74.
19. Zilles K, Amunts K (2012) Architecture of the human cerebral cortex. In J. K. Mai & G. Paxinos (Eds.), *The Human Nervous System*: 826-885. Elsevier.
20. Amunts, K. (2011). *Das Gehirn eines Sprachgenies - Eine neurobiologische Annäherung*, in *Emil Krebs. Kurier des Geistes*, P. Hahn, Editor. 2011, Oase. p. 180-203.
21. Zilles, K. & Amunts, K. (2010). Centenary of Brodmann's map - conception and fate. *Nature Reviews Neuroscience*. 11(2): p. 139-145.
22. Grodzinsky Y., Amunts K. (Eds.) (2006) *Broca’s Region*. Oxford, Oxford University Press, 419 S. (ISBN 0195177649)
23. Amunts K., Fink G.R. (2005) The convergence of Brain Structure & Function. Second Vogt Brodmann Symposium in Jülich, Germany, *Anatomy & Embryology* 210(5-6): 335-537
24. Amunts, K., Schleicher A. and Zilles, K. (2004). Outstanding language competence and cytoarchitecture in Broca's speech region. *Brain and Language*, 2004. 89(2): p. 346-353.
25. Garey, L. (2002). History of Neuroscience: Korbinian Brodmann (1868-1918). IBRO History of Neuroscience, 2002. [http://ibro.org/wp-content/uploads/2018/07/Brodmann-Korbinian.pdf]

26. Garey LJ. (1994). Brodmann's "localisation in the cerebral cortex". Garey LJ, translator/editor. London: Smith-Gordon; 1994. 2nd edn. 1999: London: Imperial College Presocietys; 3rd edn. 2006: Heidelberg: Springer.
27. Falkai, P. (1988). Hippocampal pathology in schizophrenia. A morphometric study. Thesis, Düsseldorf.
28. Sander, H.A. (1981). Morphometrische Analyse der neuropathologischen Veränderungen im Nucleus ruber bei Parkinson'scher Krankheit Thesis, Düsseldorf.
29. Herbel, W. (1979). Zur Neuroanatomie und Neuropathologie des Nucleus ruber beim Menschen. Thesis, Düsseldorf.
30. Rose JE. (1979). Korbinian Brodmann. In: Haymaker W, Schiller F, editors. The founders of neurology. 2nd edn. Springfield, IL: Charles C Thomas; 1979.
31. Dom R. (1976). Neostriatal and Thalamic Interneurons. Their role in the pathophysiology of Huntington's chorea, Parkinson's disease and catatonic schizophrenia. Thesis, Leuven.
32. Beheim-Schwarzbach, D. (1975). Weitere Untersuchungen zur cytoarchitektonischen Gliederung der Dorsalfläche der 1. Temporalwindung bei einem Sprachgenie und zwei Anthropoiden. *Zeitschrift für mikroskopisch-anatomische Forschung*. 89(5): p. 759-776.
33. Lange, H., Thörner G., & Hopf, A. (1975a). Morphometrisch-statistische Strukturanalysen des Striatum, Pallidum und Nucleus subthalamicus beim Menschen, Teil 1. J. Hirnforsch. 16, 333-350.
34. Lange, H., Thörner G., & Hopf, A. (1975b). Morphometrisch-statistische Strukturanalysen des Striatum, Pallidum und Nucleus subthalamicus beim Menschen, Teil 2. J. Hirnforsch. 16, 401-413.
35. Beheim Schwarzbach, D. (1974). Cytoarchitektonik der Dorsalfläche der 1. Temporalwindung links (T1) bei sechs menschlichen Gehirnen (darunter vier Elitegehirne) der Sammlung von C. und O. Vogt. *Zeitschrift für mikroskopisch-anatomische Forschung, 8*.
36. Hopf, A. (1969). Photometric studies on the myeloarchitecture of the human temporal lobe I. Parietal region. *Journal für Hirnforschung*,11(4): p. 253-265.
37. Sanides, F. (1962). Die Architektur des menschlichen Stirnhirns., Monogr., Ges. Neurol. Psychat. 98, 1-203, Springer, Berlin.
38. Busch, K.-T. (1960). Individuelle architektonische Differenzen der Area striata. J. Hirnforsch. 4, 535-552.
39. Schulze, H.A. (1960). Zur individuellen cytoarchitektonischen Gestaltung der linken und rechten Hemisphäre des Lobulus parietalis inferior. J. Hirnforsch. 4, 486-534.
40. Hempel, K.-J. & Treff, W.M. (1959). Die Gliazelldichte bei klinisch Gesunden und Schizophrenen. J. Hirnforsch. 4, 205-253.
41. Namba, M. (1958). Über die feineren Strukturen des medio-dorsalen Supranucleus und der Lamella medialis des Thalamus beim Menschen. J. Hirnforsch. 4, 1-41.
42. Hopf, A. (1957). Architektonische Untersuchungen an sensorischen Aphasien. *Journal für Hirnforschung*, 3(4): p. 275-530.
43. Hopf, A. and H. Gräfin Vitzthum. (1957). Über die Verteilung myeloarchitektonischer Merkmale in der Scheitellappenrinde beim Menschen. *Journal für Hirnforschung*, 3(2/3): p. 79-97.
44. Namba, M. (1957). Cytoarchitektonische Untersuchung am Striatum. J. Hirnforsch. 3, 24-48.
45. Sanides, F. (1957). Die Insulae terminales des Erwachsenengehirns des Menschen. J. Hirnforsch. 3, 243-273.
46. Buttlar-Brentano, K.v. (1956). Zur weiteren Kenntnis der Veränderung des Basalkerns bei Schizophrenen. J. Hirnforsch. 2: 271-291.
47. Hopf, A. (1956). Über die Verteilung myeloarchitektonischer Merkmale in der Stirnhirnrinde beim Menschen. *Journal für Hirnforschung*, 2(4): p. 312-333.
48. Solcher (1956). Zur Pathologie des Niger reticulatus und zu seiner Stellung im extrapyramidalen System. J. Hirnforsch. 2, 391-401.

49. Hopf, A. (1955). Über die Verteilung myeloarchitektonischer Merkmale in der isokortikalen Schläfenlappenrinde beim Menschen. *Journal für Hirnforschung*, 2: p. 36-54.
50. Buttlar-Brentano, K.v. (1954). Zur Lebensgeschichte des Ncl. basalis, tuberomammillaris, supraopticus und paraventricularis unter normalen und pathogenen Bedingungen. J. Hirnforsch. 1, 337-419.
51. Fünfgeld, E.W. (1954). Der Nucleus anterior thalami bei Schizophrenie. J. Hirnforsch. 1, 146-155.
52. Heinze G. (1954a). Zytoarchitektonische Untergliederung der Area occipitalis. J. Hirnforsch. 1: 173-198.
53. Heinze G. (1954b). Größenbestimmung und Darstellung von Rindenfeldern. J. Hirnforsch. 1: 199-205.
54. Hopf, A. (1954). Die Myeloarchitektonik des Isokortex temporalis beim Menschen. *Journal für Hirnforschung*, 1: p. 208-279.
55. Hopf, A. (1954). Zur architektonischen Gliederung der menschlichen Hirnrinde. *Journal für Hirnforschung*, 1(6): p. 442-496.
56. Hopf, A. (1954). Die Myeloarchitektonik des Isocortex temporalis beim Menschen. *Journal für Hirnforschung*, 1954. 1: p. 208-279.
57. Klatzo, I. (1954). Über das Verhalten des Nucleolarapparates in den menschlichen Pallidumzellen. J. Hirnforsch. 1, 47–60.
58. Olszewski, J., Baxter, D. (1954). Cytoarchitecture of the Human Brain Stem. Philadelphia: J. B. Lippincott Co.
59. Schiffer, D. (1954). Sur l'action reparatrice du noyau des cellules nerveuses. *J Hirnforschung*, 326-336.
60. Braitenberg G, V. (1952). Ricerche istopatologiche sulla corteccia frontale di schizofrenici. Proc. Ist. Intl. Cong. Neuropathol. Rom. Rosenberg & Sellier, Torino. Vol. 3, pp. 621-626.
61. Fünfgeld, E.W. (1952). Pathologisch-anatomische Untersuchungen im Nucleus anterior thalami bei Schizophrenie. Proc. Ist. Intl. Cong. Neuropathol. Rom. Rosenberg & Sellier, Torino. Vol. 3, pp. 648-659.
62. Wahren, W. (1952). The changes of hypothalamic nuclei in schizophrenia. Proc. Ist. Intl. Congr. Neuropathol. Rom. Rosenberg & Sellier, Torino. Vol. 3, pp. 660-673.
63. Brockhaus, H. (1942a). Zur feinen Anatomie des Septum und des Striatum. J. Psychol. Neurol. 51, 1-56. Translated in: Human Brain Dissection. Pope A. ed. (1983). U.S. Government Printing Office Publ. 381-132: 3096.Gerhardt, E. (1940). Die Cytoarchitektonik des Isocortex parietalis beim Menschen. *J. Psychol. Neurol.* 49, 367-419.
64. Brockhaus, H. (1942b). Vergleichend-anatomische Untersuchungen über den Basalkernkomplex. J. Psychol. Neurol. 51, 57-95.
65. Brockhaus, H. (1942c). Beitrag zur normalen Anatomie des Hypothalamus und der Zona incerta beim Menschen. J. Psychol. Neurol. 51, 96-195.
66. Brockhaus, H. (1938). Zur normalen und pathologischen Anatomie des Mandelkerngebietes J. Psychol. Neurol. 49, 1-136.
67. Hassler, R. (1937). Zur Normalanatomie der Substantia nigra. Versuch einer architektonischen Gliederung. J. Psychol. Neurol. 48, 1-55.
68. Rose, M. (1935). Cytoarchitektonik und Myeloarchitektonik der Großhirnrinde, in *Handbuch der Neurologie*, O. Bumke and O. Foerster, Editors. 1935, Springer: Berlin. p. 588-778.
69. Vogt, M. (1933). Reizphysiologische Untersuchungen an Säugetieren. *Journ. f. Psychol. u. Neurol. 45*(4 u. 5), 299-361.
70. Vogt, M. (1929). Über fokale Besonderheiten der Area occipitalis im cytoarchitektonischen Bilde. *J Psychol Neurol (Lpz), 39*, 506-510.
71. Vogt, M. (1928). *Über omnilaminäre Strukturdifferenzen und lineare Grenzen der architektonischen*

Felder der hinteren Zentralwindung des Menschen. Retrieved from http://resolver.hebis.de/retro/a3330505

72. Rose, M. (1928). Die Ontogenie der Inselrinde. *Journal für Psychologie und Neurologie*, 86(3 u. 4): p. 182-209.
73. Rose, M. (1928). Die Inselrinde des Menschen und der Tiere. *Journal für Psychologie und Neurologie*, 37: p. 467-624.
74. Rose, M. (1927). Die sog. Riechrinde beim Menschen und beim Affen. II. Teil des "Allocortex bei Tier und Mensch". *Journal für Psychologie und Neurologie*, 34: p. 261-401.
75. Rose, M. (1927). Gyrus limbicus anterior und Regio retrosplenialis. *Journal für Psychologie und Neurologie*, 35(3 u. 4): p. 65-173.
76. Rose, M. (1927). Allocortex bei Tier und Mensch. Die sog. Riechrinde beim Menschen und beim Affen. *Journal für Psychologie und Neurologie*, 34: p. 261-401.
77. Rose, M. (1926). Über das histogenetische Prinzip der Einteilung der Großhirnrinde. *Journal für Psychologie und Neurologie*, 32(3): p. 98-160.
78. Rose, M. (1926). Der Allocortex bei Tier und Mensch I. Teil. *Journal für Psychologie und Neurologie*, 34(1 u. 2): p. 1-111.
79. Wetzel, G. (1923). Hirnwindungen und Brodmann-Vogtsche Felder in ihren gesetzmäßigen Beziehungen. *Journal für Psychologie und Neurologie, 29*, 434.
46. Brodmann, K. (1914). Physiologie des Gehirns. In: F. Krause (Ed.) Die allgemeine Chirurgie der Gehirnkrankheiten. Erster Teil, zweiter Abschnitt. Stuttgart: Enke. Pp 85-426.
47. Brodmann, K. (1913). Neuere Forschungsergebnisse der Großhirnrindenanatomie, mit besonderer Berücksichtigung anthropologischer Fragen. *Verh Ges Dtsch Naturf Ärzte* 35: 200-240.
48. Brodmann, K. (1912). Neue Ergebnisse über die vergleichende histologische Lokalisation der Grosshirnrinde mit besonderer Berücksichtigung des Stirnhirns. *Anat Anz*, 41(Suppl), 157-216.
49. Brodmann, K. (1910). Feinere Anatomie des Grosshirns. In Handbuch der Neurologie: Erster Band: Allgemeine Neurologie (pp. 206-307). Berlin, Heidelberg: Springer Berlin Heidelberg.
50. Brodmann, K. (1909). Vergleichende Lokalisationslehre der Grosshirnrinde in ihren Prinzipien dargestellt auf Grund des Zellenbaues. Leipzig: Barth.
51. Brodmann, K. (1908a). Beiträge zur histologischen Lokalisation der Grosshirnrinde. VII. Mitteilung: Die cytoarchitektonische Cortexgliederung der Halbaffen (Lemuriden). *J Psychol Neurol* 10 287-334.
52. Brodmann, K. (1908b). Beiträge zur histologischen Lokalisation der Grosshirnrinde. VI. Mitteilung: Die Cortexgliederung des Menschen. *J Psychol Neurol* 10 231-246.
53. Brodmann, K. (1908c). Über Rindenmessungen. *Zbl Nervenhk* 31/N.F. 19: 781-798.
54. Brodmann, K. (1907). Bemerkung über die Fibrillogenie und ihre Beziehungen zur Myelogenie mit besonderer Berücksichtigung des Cortex cerebri. *Neurol Zbl* 26: 338-349.
55. Brodmann, K. (1906). Beiträge zur histologischen Lokalisation der Grosshirnrinde. Fünfte Mitteilung: Über den allgemeinen Bauplan des Cortex pallii bei den Mammalieren und zwei homologe Rindenfelder im Besonderen. Zugleich ein Beitrag zur Furchenlehre. *J Psychol Neurol* 6 275-400.
56. Bielschowsky M, Brodmann K. (1905). Zur feineren Histologie und Histopathologie der Großhirnrinde mit besonderer Berücksichtigung der Dementia paralytika, Dementia senilis und Idiotie. *J Psychol*, 5: 173–199.
57. Brodmann, K. (1905a). Beiträge zur histologischen Lokalisation der Grosshirnrinde. Vierte Mitteilung: Die Riesenpyramidentypus und sein Verhalten zu den Furchen bei den Karnivoren. *J Psychol Neurol* 6 108-120.
58. Brodmann, K. (1905b). Beiträge zur histologischen Lokalisation der Grosshirnrinde. Dritte Mitteilung: Die Rindenfelder der niederen Affen. *J Psychol Neurol* 4 177-226.
59. Brodmann, K. (1904). Experimenteller und klinischer Beitrag zur Psychopathologie der polyneuritischen Psychose, B Experimenteller Teil. *J Psychol* 4: 1-48.

60. Brodmann, K. (1903a). Experimenteller und klinischer Beitrag zur Psychopathologie der polyneuritischen Psychose, A: klinischer Teil. *J Psychol* 1: 225-246.
61. Brodmann, K. (1903b). Pletysmographische Studien am Menschen. Erster Teil. Untersuchungen über das Volumen des Gehirns und des Vorderarms im Schlafe. *J Psychol* 1: 10-71, 84-88
62. Brodmann, K. (1903c). Beiträge zur histologischen Lokalisation der Grosshirnrinde. Zweite Mitteilung: Der Calcarinatypus. *J Psychol Neurol* 2 133-159
63. Brodmann, K. (1903d). Beiträge zur histologischen Lokalisation der Grosshirnrinde. Erste Mitteilung: Die Regio Rolandica. *J Psychol Neurol* 2 79-107.
64. Brodmann, K. (1901). Die Anwendung des Polarisationsmikroskops auf die Untersuchung degenerierter markhaltiger Nervenfasern. *Zbl Nervenhk* 24/N.F. 12: 193-213.
